# The influence of urban form on the grid integration of renewable energy technologies and distributed energy systems

**DOI:** 10.1038/s41598-019-53653-w

**Published:** 2019-11-28

**Authors:** A. T. D. Perera, Silvia Coccolo, Jean-Louis Scartezzini

**Affiliations:** Solar Energy and Building Physics Laboratory (LESO-PB), Ecole Polytechnique Fédérale de Lausanne (EPFL), CH-1015 Lausanne, Switzerland

**Keywords:** Power stations, Solar cells

## Abstract

Standard and newly designed building blocks for complex urban sites– also designated by urban archetypes - are used in this study to quantify the influence of urban forms on their energy demand and energy systems design. An energy hub, which consists on a multi-carrier energy system involving multiple energy conversion, storage and/or network technologies, is employed to quantify the impact of the urban morphology on the energy system requirements. This study reveals that urban archetypes have a notable influence on the heating and cooling energy demands of city districts that can be characterized using form factors and floor area ratio. However, the influence on demand profiles cannot be assessed based on the aforementioned indicators. The cost of energy systems can increase up to 50% due to the impact of urban forms that are well beyond the increase of peak and/or annual energy demands. In addition, renewable energy integration to the grid as well its utilization in districts is influenced by urban forms. This makes it essential to consider energy system design as a part of the urban planning process moving even beyond building simulation.

## Introduction

The sustainability of cities regarding energy has been widely discussed in recent years and even included into the UN Sustainable Development Goals^1^. However, no satisfactory method to quantify the impact of the complex chain of reactions that influence urban energy infrastructures has been presented to date.

The physical interactions among buildings in the urban environment play a vital role when considering the energy demand in cities. According to Baker and Steemer^2^, five factors leverage building energy performance in the urban context, i.e. urban climate, urban morphology, building physics, Heating Ventilation and Air-Conditioning (HVAC) systems and occupant behavior. Energy efficiency, as well as the sustainability of cities, have been addressed from different perspectives^3^. Among these, two complementary approaches can be clearly outlined when referring to the most recent literature:

(i) Increasing the energy efficiency of buildings and districts, as well as fostering sustainable energy technologies in urban infrastructures, by assessing the potential of renewable energy technologies, designing and sizing them in an optimal way, improving their operation in urban energy systems, though the renovation of the building stock;
(ii) Enhancing the energy and environmental performance of urban forms through an efficient urban planning that mitigate the impact of urban microclimate and climate change on districts and cities.

Both methodologies play a vital role in improving the sustainability of cities as demonstrated in detail in the recent literature^4,5^. When it comes to the first approach, Perera *et al*.^6^ considered the potential of distributed energy systems, such as energy hubs, to integrate renewable energy technologies into the urban infrastructure. Fazlollahi *et al*.^7,8^ introduced a novel method to optimize urban energy systems combining several distributed energy systems with the distribution network. Having a better understanding of the energy demand is essential when designing distributed energy systems. Fonseca *et al*.^9,10^ introduced City Energy Analysis to design urban energy systems carefully considering the energy demand at building scale using statistically representative urban archetypes. Le Guen *et al*.^11^ developed a computational platform that considers the energy demand in detail including interactions among buildings when designing distributed energy systems: this platform is used to obtain the optimal levels of building renovation and energy system improvements. Perera *et al*. assessed the impact of urban mirco-climate on energy system design^12^. All these studies are focused on improvements considering both buildings and energy systems. However, none of them considered the influence of urban forms; hence, it is difficult to directly extend such methods to urban planning in a concrete manner. In addition, the mutual interactions among buildings have not been considered in most of the studies, although these significantly influence the energy efficiency and renewable energy integration. Hence, according to Salat^13^, it is important to move beyond building level to consider urban form, which can notably reduce the energy consumption of buildings and foster renewable energy integration in districts and cities.

From the perspective of urban form and morphology, the debate is open, to define the “best” urban form in terms of sustainability of the urban environment. The discussion on the “compact city” versus the “dispersed city”^14,15^ can be brought as one example. A sustainable urban planning plays a leading role in optimizing urban energy consumption. Ratti *et al*.^16^ outlined the relationship between energy consumption in cities and urban texture and related its influence on energy efficiency. Different attempts have been made to quantify the influence of urban forms on the energy demand of buildings. Liu *et al*.^17^ evaluated the impact of urban density on heat transfer among building stocks simply considering the distances between buildings; however, such a method cannot be applied to complex urban morphologies. Ratti *et al*.^16^. introduced the concept of urban archetypes (i.e. building blocks for complex urban forms that can be observed in a city), which is elaborated in detail in the next section, in order to handle the complexity of urban morphology. Okeil *et al*.^18^ evaluated the effectiveness of harnessing solar energy in different urban forms introduced by Ratti *et al*.^16^, Sanaieian *et al*.^19^ evaluated the impact of urban forms on thermal performance, solar access and ventilation of buildings using urban archetypes while Taleghani *et al*.^20^ evaluate the impact of the latter on outdoor thermal comfort. These studies clearly reflect that the urban form has a notable impact on the energy demand of buildings and that urban archetypes can be used to get a better understanding of the impact of urban forms on the related energy performance. However, none of these studies focuses on the impact of urban archetypes on renewable energy integration to the grid as well as energy systems design.

The two approaches mentioned above (e.g. improving the energy efficiency of urban forms, improving the energy efficiency at the building scale and integrating renewable energy technologies) are adopted in this study to foster the energy efficiency and sustainability at the urban scale: combining all of them can speed up the energy transition in the urban sector. Towards this end, the study focuses on quantifying the impact of urban forms on energy demand as well as its consequences for energy systems design, which can lead to improved energy sustainability in future cities through an optimal urban planning. The urban morphology can be very complex in certain instances and might be difficult to represent using simple archetypes. To handle this issue, integrated urban archetypes are introduced by extending the modular archetype defined by Ratti *et al*.^16^. Towards addressing the main research issue, the design of distributed energy systems is optimized considering the energy demand of a selected set of urban archetypes. Multi-objective optimization programming using mathematical optimization methods involving more than one objective function was used for that purpose. The cost and the energy system autonomy with regard to the grid were considered to optimally design the energy system. Subsequently, the impact of the urban form on cost, autonomy and renewable energy integration level to the grid are evaluated for both simple and complex urban archetypes to assess the influence of urban forms on the urban energy infrastructure.

## Urban Archetypes

**Figure 1:**
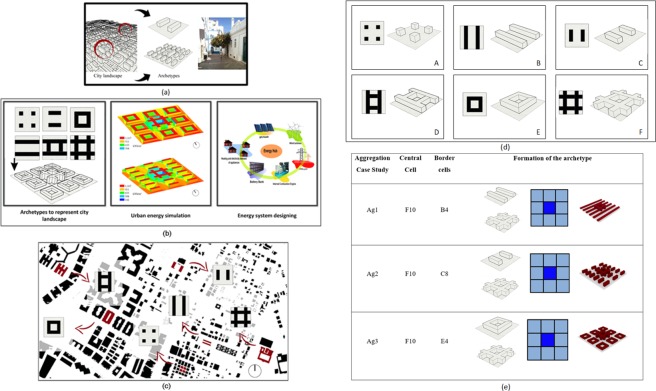
(**a**) presents the concept of archetype which represent a typical neighborhood in terms of geometric and volumetric properties when working at the urban scale. (**b**) presents the workflow of the study where the concept of archetypes is combined with urban energy simulation and energy system designing process to understand the influences of urban form on energy system design process (this is an extension to the figure appear in ref. ^6^). (**c**) presents the urban archetypes, being part of the current urban design in the city of Dubai. (**d**) presents the modular archetypes used for the study. (**e**) presents the integrated urban archetypes.

An urban archetype (Fig. 1) is a profile of an individual neighborhood that represents a synthesis of several environmental and architectural parameters commonly used to describe an urban environment. Urban archetypes can be used to represent a typical neighborhood in terms of geometrical and volumetric properties at the urban scale^21^. Several studies employed the latter to investigate the relationship between the urban forms and environmental variables, such as the availability of solar energy and daylight within city districts^16,21,22^. Six urban archetypes adapted from Ratti^21^ were selected in this study; these urban configurations are comparable to building blocks of complex urban morphologies that can be observed in a city. The parameters which are characterizing the urban archetypes are: (i) the Ground Floor Area (m^2^), (ii) the floors number (−), (iii) the Treated Floor Area (m^2^), (iv) the Sky View Factor (−), (v) the Floor Area Ratio (−), (vi) the Site Coverage (%) and (vii) the Form Factor (−). The Sky View Factor (SVF) is defined as the ratio between the radiation received by a planar surface and the one from the entire hemispheric radiating environment. The Floor Area Ratio (FAR) is equal to the ratio of the building gross floor area to the site area. The Site Coverage (%) is defined as the ratio of the buildings footprint to the site area. The Form Factor (FF) is equal to the ratio of the external building envelope versus its gross floor area. It represents a ratio, which is inversely proportional to the thermal losses of the envelope. As an example, taking two buildings located in temperate climate, with the same envelope types, the edifice with an FF higher than one will face more thermal losses, and consequently higher energy demand, than the one with an FF lower than one. Figure 1(a–d) shows the suggested archetypes and their location within the city of Dubai; indeed, the considered block geometries are already part of the urban texture of the city. One should notice that their urban characteristics vary as a function of their dimensions (length, width and height), as well as the mutual interrelations between the city blocks.

A possible development of urban archetypes are the modular ones, which represent buildings in a simple neighborhood. It is however important to understand the impact of the scaling-up process when moving from a simple neighborhood to the urban scale: both horizontal and vertical growth must be considered in this context. Vertical growth corresponds to adding more floors to an existing modular archetype, which will increase the treated floor area of the buildings while maintaining the same ground floor area and site coverage. In contrast, an horizontal expansion extends the ground floor area while maintaining the number of floors. The planar dimensions of a modular archetype remain identical in case of a vertical expansion. Similarly, an horizontal expansion can be achieved by increasing the planar dimensions of the modular archetype using a constant factor. However, it is difficult in reality to observe such a proportionate expansion of modular archetypes when considering an horizontal growth: it is generally observed in this case that different modular archetypes are mixed together to build up complex urban forms.

This study considers the evolution of modular archetypes from simple neighborhoods to complex urban forms through both vertical and horizontal expansions. Ten different cases were envisaged for each modular archetype as a function of the floors number in order to assess the impact of a vertical growth. In the case of an horizontal growth, this study considers a grid that integrates different urban archetypes based on modular ones. The grid consists of nine cells, combining the modular archetype of Fig. 1(e), the central one corresponding being the highest building (e.g. with 10 floors). The cells around are defined by the way of the other case studies, the main objective being to reach a total floor area of 145’000 m^2^ with a tolerance lower than 5’000 m^2^ and a relative difference lower than 5%. Three different configurations were finally chosen. Their geometrical features are given in Table A1 (see Supplementary Information); their total floor area is equal to 147’000 m^2^.

### Designing energy systems for archetypes

A computational platform consisting of several computational tools was employed to design urban energy systems for each archetype: an overview of the latter is given in ref. ^11,13^. The computational platform includes a GIS database (QGIS) of the urban site, the building height, the year of construction, etc. The meteorological data for the site location (such as wind speed, solar irradiation, ambient temperature, etc.) used for the computer simulations as well as the energy systems optimization was extracted from Meteonorm data. Two locations, showing very different climatic conditions were chosen: the village of Hemberg in Switzerland and the city of Dubai in the United Arab Emirates. The urban simulation software CitySim Pro was used to assess the energy performance of the different buildings of the urban site for each archetype. All the thermo-physical characteristics of the buildings, such as the U-values of the opaque envelope materials and the Solar Heat Gain Coefficients (SHGC) of the windows, the air infiltration rate, the occupancy profile, the outdoor materials features, as well as the urban environment were introduced within CitySim Pro. A detailed description of the urban simulation model relying on CitySim Pro is given in the Methodology section. The energy demand profiles of the archetypes assessed by the urban simulation model were used to design the urban energy systems. An energy hub that caters the heating, cooling and electricity demand of the urban site is considered in this study. The hub is operated in a grid connected mode both selling and purchasing electricity to and from the grid while accommodating fluctuations in demand and generation. It consists of renewable energy technologies, a dispatchable energy source as well an energy storage facility. Solar photovoltaic panels (PV panels) and wind turbines are both considered in the energy hub, which are non-dispatchable energy sources, meaning that the power generation varies with the changes in wind and solar energy availability. An Internal Combustion Generator (ICG) is used as dispatchable energy source, helping to absorb the fluctuations of the electricity demand along with the battery bank. Heating and cooling demands are catered by the way of an heat pump and air-conditioners respectively. The configuration of the hub depends on the energy demand of the archetype obtained using the urban simulation software. The computer model presented in the Methodology section is used to build-up the optimal energy system for the archetype.

### Influence of urban form on the energy demand

**Figure 2:**
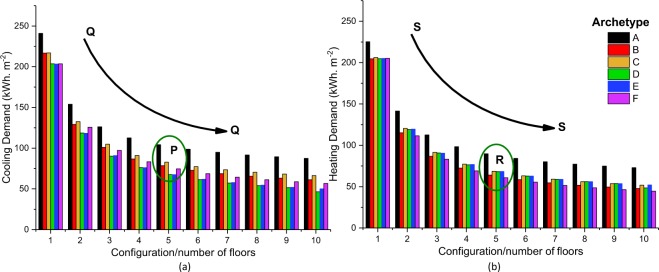
Annual cooling and heating demand respectively for Dubai and Hemberg are presented in (**a**,**b**). Lines Q-Q and S-S show that annual energy demand for cooling and heating generally decreases with an increase in the number of floors. Circle P shows that there is a significant difference in energy demand per unit area (even for the same number of floors) for the case of Dubai, which is lower in Hemberg as shown in circle.

The annual heating and cooling energy intensity (e.g. energy demand per unit of floor area), assessed for Dubaï and Hemberg, is shown resp. in Fig. 2(a,b). Both Q-Q and S-S curves show that there is a significant drop in the energy intensity when increasing the number of floors. For instance, the annual cooling intensity decreases from 240 to 99 kWh/m^2^ when moving from archetype A1 to archetype A6 in the Dubai case study; a similar decrease in energy intensity can be observed for Hemberg. However, there is a significant variation in the cooling demand between different urban archetypes having the same number of floors, as illustrated by the circle P of Fig. 2(a): the cooling demand decreases by 35% from 104.35 to 67.58 kWh/m^2^ when moving from archetype A5 to archetype D5, even if they have the same number of floors. This clearly highlights that archetypes can have a significant impact on the energy demand. However, the impact is lower for Hemberg as shown by comparing the results within the circles P and R.

**Figure 3:**
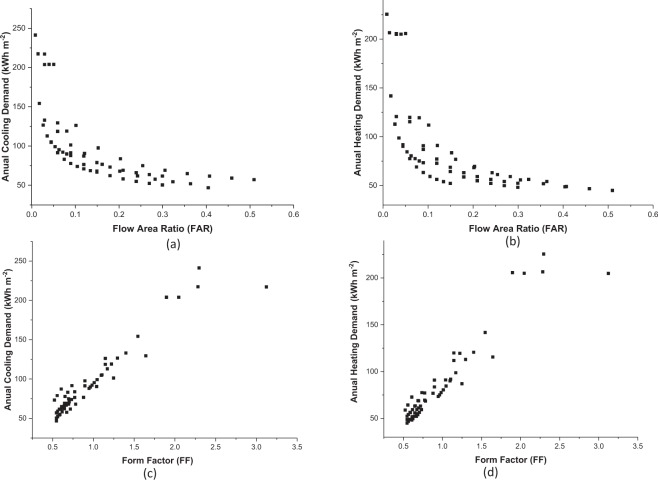
Variations of (**a**) cooling and (**b**) heating demand for Dubai and Hemberg with Floor Area Ratio (FAR) are presented in (**a,b**). Variations of cooling and heating demand for Dubai and Hemberg with Form Factor (FF) are presented in (**c,d**).

The variation of the cooling and heating intensities in regards to the Floor Area Ratio (FAR) and the Form Factor (FF) is comparable for Dubai and Hemberg (Fig. 3(a–d)). The demand shows a substantial decrease with the FAR while it increases linearly with the FF for both locations. For instance, increasing the Form Factor from 0.55 (Case Study F10) to 1.18 (Case Study A4), leads to a 100% increase of heating demand from 44.6 to 98.3 kWh·m^−2^ for Hemberg. A smaller FF leads accordingly to a lower heating intensity, a smaller external envelope area reducing the thermal conductive and convective losses of the building, which results in a lower heating demand. This specific aspect is also reflected in the FAR, larger values corresponding to a more compact building leading to a lower heating demand. However, Fig. 3(b) shows that the urban archetypes with a similar heating demand (around 205 kWh·m^−2^) can have very different Form Factors, passing from 1.9 (case study E1) to 3.1 (case study B1). This reflects that both FF and FAR only provide a general trend, the urban forms having a larger on the heating intensity for some cases. Hence, it is important to consider the influence of hourly demand profiles to get a broader idea of the impact of the urban morphology on the energy system with regard to the design process.

### Influence on the hourly energy demand

**Figure 4:**
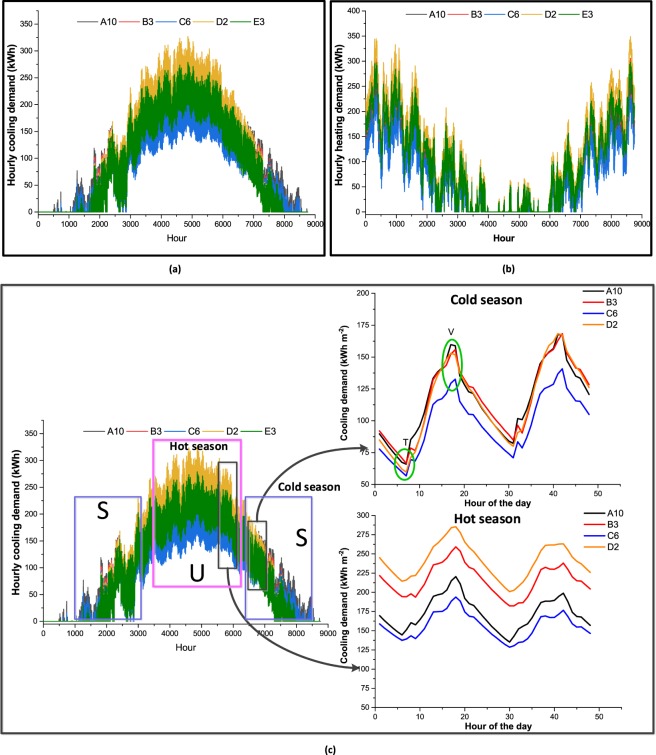
Hourly variation of cooling (**a**) and heating demand (**b**) respectively for Dubai and Hemberg case studies. (**c**) A detailed analysis of the hourly cooling demand profile of Dubai. Rectangles U and S present the demand during summer and autumn and spring respectively. Frames 1 and 2 are used to further analyze the demand profile.

The next step is to consider the entire set of urban archetypes used so far to analyze the different demand profiles on an hourly scale (Fig. 4). For this purpose, five archetypes were selected (A10, B3, C6, D2 and E3) from the entire set of 60 archetypes, representing different modular types with different numbers of floors. However, the selected archetypes have a total floor area close to 9’000 m^2^, meaning that the corresponding energy systems can be compared in order to highlight the impact of the urban morphology.

Hourly cooling and heating profiles for Dubai and Hemberg are illustrated in Fig. 4(a,b). The demand profiles for both locations show complex variations at the hourly level making their analysis more challenging. Hence, the hourly cooling demand profile for Dubai is considered and divided into three sections marked by colored rectangles on Fig. 4(c). The blue rectangles marked as S provide a similar variation in demand, which differs from the purple rectangle marked as U. Subsequently, two frames are taken and enlarged, each presenting a time span of 48 hours. Frame 1 presents a typical distribution of the cooling demand during summer (Hot season) while frame 2 presents the demand profile during autumn and spring (Cold season).

Following the variations observed in the annual energy demand (as shown in Fig. 2), it is expected that the hourly demand profile will shift down according to the annual demand. Such a clear shift in the demand profile can be observed for the Cold season (Fig. 4(c), Frame 2). A clear separation of the demand profiles can be observed in this context with relatively less fluctuations in the profile. However, when moving to Hot season (Fig. 4(c), Frame 1), a few significant changes can be observed. First, it is no longer possible to differentiate the demand profiles in the Hot season compared to the Cold one. In certain instances, a significant difference in cooling demand can be observed when considering C6 in comparison to the other archetypes (as marked by Circle V in Frame 2), while they are very close to each other in other instances (as marked by circle T in Frame 2). When considering the Hot and Cold seasons, it can be concluded that the difference in the energy demand is quite negligible in valleys (as marked in circle T) compared to the energy demand during peak periods, as observed in the Hot season. As a result, energy systems designed for archetypes such as D2, for instance, need to operate at very low partial loads compared to the energy systems installed in C6; this can have a significant impact on their design, especially when considering life cycle cost, environmental impact and autonomy level.

### Impact of the urban archetypes on the energy system design

The hourly variation in renewable energy availability makes its integration process more challenging, especially when designing distributed energy systems while maintaining system autonomy. Therefore, keeping the energy autonomy at building and neighborhood scale is considered as a main priority for renewable energy integration. Grid integration (GI) is an indicator which presents a better overview about the autonomy (GI is defined in the Methodology section). Similarly, the financial aspect of the energy system is reflected by the Net Present Value (NPV) (NPV is defined in the Methodology section). It is difficult to optimize these conflicting objectives simultaneously. The Pareto front in multi-objective optimization methods gather the optimal set of solutions considering both conflicting objectives; when moving from one Pareto solution to another one, an objective will be improved while there is a ‘better’ compromise in the first Pareto solution.

**Figure 5:**
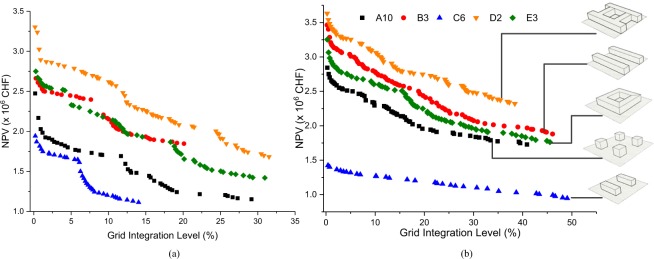
Pareto fronts for five urban forms considering NPV and Grid Integration level for the climate of (**a**) Hemberg and (**b**) Dubai (R). A higher NPV indicates a higher cost while lower GI indicates higher autonomy.

The Pareto fronts obtained considering the NPV and the autonomy level for the selected urban archetypes and the meteorological conditions of Hemberg are presented in Fig. 5(a). The latter show that an increase of NPV is induced by the minimization of the grid integration level for sake of the system autonomy. The Pareto fronts of B3 and E3 evolve in a quite close manner. Beside these two cases, a clear shift in the Pareto fronts can be observed when moving from one urban form to another. For instance, the Pareto solutions show a cost lower than 2 million CHF for the urban form C6; it is larger than 3 million CHF for the urban form D2, when reaching the stand-alone condition (e.g. GI = 0%), which represents a substantial cost increase. More importantly, the shape of the Pareto fronts shows a significant difference, when moving from one urban form to another. A sudden drop in cost can be observed for the GI levels of A10 and D2 corresponding to the standalone condition (fully autonomous); a more gradual decrease in cost can be observed for other urban forms. The Pareto fronts reveal accordingly that urban forms have a notable impact on the energy system design as well as on the grid integration level.

Besides a qualitative analysis, it is important to quantify the influence of the urban form from a cost and system autonomy perspective. When looking at C6 and A10 Pareto fronts for the village of Hemberg in Switzerland (Fig. 5(a)), it is obvious that both reach the same NPV level in an asymptotic way when increasing the grid interactions. However, there is a significant difference in the grid interaction levels for the same costs. The archetype C6 maintains a 14% grid integration level at a cost of 1.1 million CHF, whereas A10 presents an integration level of 29% for the same cost. This clearly shows that a higher autonomy level can be maintained for a much lower price for some urban configurations. Furthermore, a grid integration level of 10% can be reached for 1.2 million CHF for archetype C6, whereas the cost reaches 2.6 million CHF for archetype D2 at the same integration level. In brief, urban forms have a ‘price to pay’ from an energy system perspective.

It is also interesting to assess the impact of climate on the energy efficiency of the urban form. Moving from Hemberg (CH) to Dubai (UAE), on can observe that the Pareto fronts follow the same pattern when arranged in the order of increasing cost (Fig. 5(b). However, the shape of the Pareto fronts is more homogenous for Dubai. A sudden drop in NPV can be observed when increasing the grid integration level starting from a stand-alone status (e.g. GI = 0%), followed by a gradual cost reduction when further increasing the grid integration level. However, a significant cost difference can be observed when comparing from archetype C6 to A10, which is not observed for Hemberg. Furthermore, the two Pareto fronts corresponding to B3 and E3 are clearly separated for Dubai due to the difference in cooling demand; this is not the case for Hemberg where the two curves are superimposed and lead by the heating demand. In general, noticeable changes are observed in the Pareto fronts of Hemberg and Dubai, although the ranking of the Pareto fronts according to the NPV level is identical. In conclusion, it can be stated that the energy consumption of buildings for heating and/or cooling depends on the urban form, and may lead to notable differences of the energy demand profiles for one location or another; this induces also notable changes for the urban energy system when considering different autonomy levels.

### Impact of the energy demand on energy system design and operation

**Figure 6:**
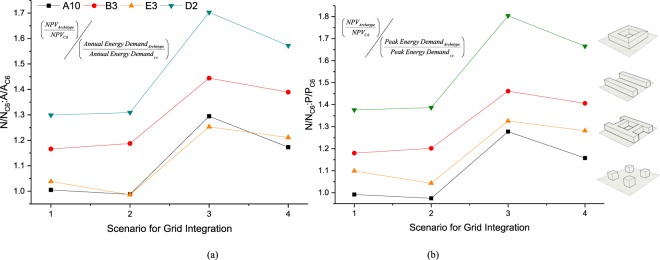
Variation of N/N_C6_:A/A_C6_ and N/N_C6_:P/P_C6_ for different scenarios of grid integration are respectively presented in (**a,b**). Both figures clearly show that except for two scenarios in A10 the ratio of NPV of the energy system is greater than the ratio of annual and peak energy demands and presents a complex variation. More importantly, the impact of urban form on the energy system infrastructure cost might be well above the values that we can gain through an urban energy simulation such as peak and energy demand.

Urban forms can influence both peak demand and demand patterns, which may lead to a notable difference in the annual energy consumption of buildings and varying demands on the energy system, as well as a notable cost differences for the same autonomy level. It is important to assess whether increases in peak or annual demand have an impact on the cost of an energy system in a similar manner. Four Pareto solutions were considered for each urban form having a similar grid interaction level. The NPV of these Pareto solutions were divided by the NPV of archetype C6 (e.g. NPV/NPV_C6_), this urban form showing the lowest peak and annual energy demand for both locations. Similarly, the ratios of the peak demand of a urban form by the peak demand of archetype C6 (e.g. P/P_C6_) as well as the annual energy demand (e.g. A/A_C6_) where computed, as illustrated on Fig. 6. The quotient of NPV/NPV_C6_ divided by P/P_C6,_ as well as NPV/NPV_C6_ divided by A/A_C6_, are computed in order to compare the impact of variations in peak and annual demand on the urban energy system, as a result of a modification of an urban form.

Figure 6(a,b) show that both (NPV/NPV_C6_) / (A/A_C6_) and (NPV/NPV_C6_) / (P/P_C6_) ratios have values larger than one, except for grid integration scenari of archetypes A10 and E3. This indicates that the cost of the system increases in a non-linear way relative to the peak and annual demand, for other grid interaction scenarios than the one of archetype C6. More importantly, both ratios reach 1.8 for archetype D2 in Scenario 3, which indicates that there is a drastic costs increase well beyond the increase in annual or peak demand. This clearly highlights the importance of evaluating the sensitivity of energy systems to the urban form. When analyzing the demand profile of archetypes A10, B3, E3 D2 and C6, D2 shows the highest peak demand. Nonetheless, the demand requirements when in absence of cooling or heating (which corresponds to the minimal demand) is identical for all scenarios (since the demand for appliances is supposed to be equal). Hence, demand fluctuation in D2 is significant compared to the other scenarios, resulting in a non-linear cost increase relative to the peak demand and/or the annual demand. The same argument can be advanced to explain the fluctuations for the three other urban forms. Hence, it is clear that the influence of an urban form cannot be inferred using buildings and/or urban simulations, which requires to account for energy system design in urban planning.

### Renewable energy integration at building and system level

**Figure 7:**
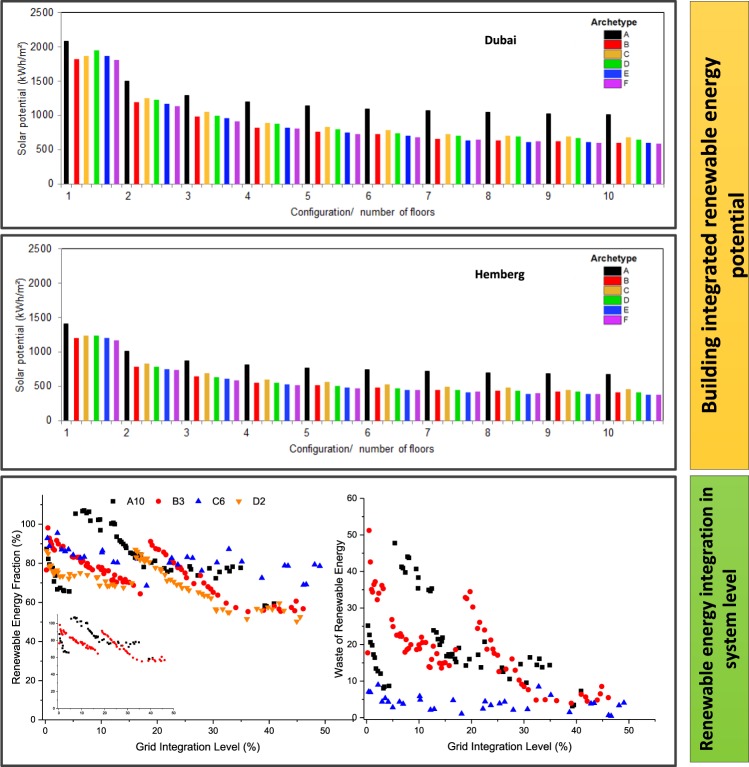
Presents the renewable energy integration process. Two figures at the top presents the potential for solar PV generation at the building scale. Renewable energy integration into Archetypes. Annual solar irradiation per unit floor area for different urban forms for Dubai and Hemberg are presented in this figure. The two graphs right on the bottom demonstrate renewable energy integration at system level. Fluctuations in the renewable energy fraction (left) and Waste of Renewable Energy (WRE) for the Pareto solutions considering NPV and grid Integration level as objective functions (right) for Dubai case.

Roofs receive more solar radiation than facades and are therefore often preferred for Building integrated Photovoltaic (BiPV) power plants for financial and esthetical reasons. Nonetheless, integration of PV panels into facades is rapidly growing due to the reduction PV panels costs, as well as the important related potential at the urban scale. Therefore, cumulative solar radiation on both facades and roofs was considered in this study. The annual solar energy per unit floor area captured by the building envelope is plotted for 60 different urban forms for Hemberg and Dubai (Fig. 7). As one may observe, the maximal amount of solar energy received reaches 1,402 kWh/m^2^ and 2,085 kWh/m^2^ respectively in the A1 configuration for Hemberg and Dubai. For the E10 configuration, the minimal solar energy received reaches 370 kWh/m^2^ and 591 kWh/m^2^. The roof area remains constant while the number of floors increases: this explains why the solar energy received per unit floor area decreases with the number of floors for the same urban form. However, the most important fact is the sensitivity to urban form of the solar energy potential. Significant changes can be observed among the different urban forms. The difference is of a factor two between archetypes A and E. It can be concluded that the urban form has a notable impact on the solar energy potential at building level.

Besides a qualitative assessment of the solar energy potential for BiPV technologies, it is also important to assess the provisions for renewable energy integration at the urban system level. Characteristics of the demand profile are of notable influence in this context and the latter depend on the urban form as discussed previously. The renewable energy generation fraction of archetypes A10, B3 and E3 decreases down to a certain value with an increase of the grid integration level and suddenly jumps up again (Fig. 7(c)); afterwards it starts to decrease again, but apparently without a common pattern except for B3 and D2 (the sudden jump up is due to the change in the generator capacity which can be understood by going through^23,24^). A gentle decrease in the renewable energy generation fraction can be observed considering the Pareto solutions of archetype C, which is totally different from the other three. In general, the fluctuations of renewable energy contribution with respect to grid integration level are significantly influenced by urban forms. When accounting for the renewable energy generation for all four urban forms, an optimistic picture can be drawn. Renewable energy generation can overpass the demand in certain circumstances (e.g. A10), leading to an excess of electricity injected to the grid. The renewable energy generation corresponds to 50–60% of the annual demand for the minimal NPV in regards to archetypes A10, B3 and C6. It ranges between 70% and 80% for the archetype C, which corresponds to a 20% increase in renewable energy generation fraction compared to the others. In general, irrespective of the urban form, energy hubs can incorporate renewable technologies up to 50% of the annual energy demand.

### From modular archetypes to cities

**Figure 8:**
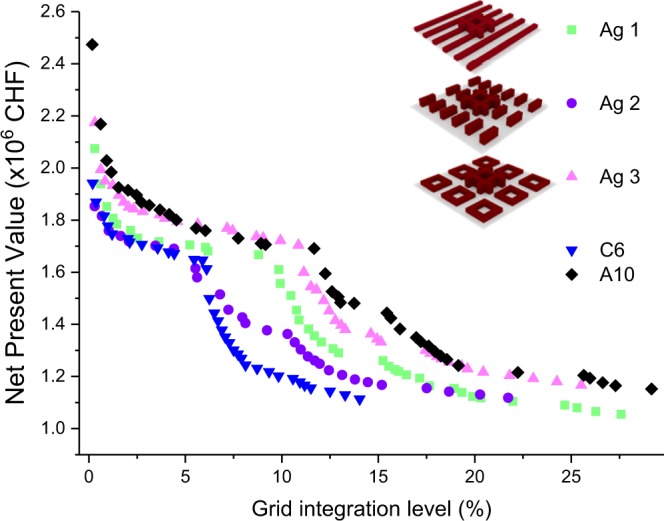
Comparison of the Pareto fronts obtained for integrated urban archetypes. The Pareto fronts of the aggregated urban forms move quite close to each other when compared to the Pareto fronts of the two modular archetypes. Aggregated Urban forms cover a much larger floor area. In order to avoid this mismatch, the energy demand profile is normalized considering the floor area. This implies that the aggregated archetype is catered using several energy systems (each having a uniform demand profile) where each energy system is focused on catering the demand of buildings with a floor area of 9000 m^2^.

Three Pareto fronts generated for the three aggregated urban archetypes, combining the simple urban forms C6 and A10, are presented in Fig. 8. When developing the aggregated archetypes, the modular archetypes are taken in a way to present the vertical and horizontal expansion along with the increase in urban complexity as discussed in^25^ The Pareto fronts show a significant drop in NPV compared to archetypes D2 and E3. This is due to the fact that the basic urban forms were more densified by building up aggregated urban forms, reducing the energy demand per unit floor area. The most important fact is that a significant change in NPV is not observed among the aggregated archetypes. The three Pareto fronts corresponding the aggregated archetypes (e.g. Ag1, Ag2 and Ag3) are comprised in between those of the simple urban forms C6 and A10. This reflects the fact that aggregated urban forms combining different simple urban forms are tending to behave differently than the original urban forms. More importantly, no significant difference in the energy system is observed for aggregated urban forms. However, this specific point needs to be further studied by deriving more aggregated urban archetypes using simple urban forms and subsequently implementing them in real world situations.

## Conclusions and Perspectives

This study reveals that urban forms have a notable impact on the energy demand of city districts. The annual demand can increase by 35% due to the urban form in the case of the village of Hemberg (Switzerland) accounting for the six different urban archetypes considered in this study. These results are strongly influenced by the climatic conditions. Although performance indicators, such as the Form Factor (FF) and the Floor Area Ratio (FAR) can be used to get an overview about the influence of urban forms on the energy demand, the latter do not provide a detailed information. Analyzing the hourly energy demand of the different archetypes allows to get an overview about the influence of urban forms on the demand profile, which can be splitted in two parts. A direct shift (clear separation) in demand profile can be observed among the archetypes for a yearly period, while the rest remains very close to each other. As a consequence, a significant fluctuation in demand profile can be observed for some archetypes notably influencing the energy system design. As a result, the autonomy level of the system can be improved by 10–15% by selecting an optimal urban form, and more importantly, the cost of the energy system can be reduced by 30–50% through an appropriate urban planning.

The influence of urban forms was further examined by focusing on the renewable energy integration perspective. As a consequence of the variation in demand profile due to urban forms, the waste of renewable energy notably changes. Except for one (C6), all archetypes have a larger waste of renewable energy during fully autonomous operations: it can reach up to half of the annual demand in certain instances. The study also shows that urban forms have an impact on the integration of renewable energy technologies, such as BiPV for instance. An optimal selection of the urban form can maximize the on-site renewable energy generation. In general, the urban form has a significant impact on the renewable energy integration. When comparing the two cities it is observed that a notable change in the NPV is observed when moving from Dubai to Hemberg. Nonetheless, the impact of urban configuration on the energy system is visible irrespective of the location. Archetype C6 performed well for both the locations when compared to the other archetypes which resulted in a significant improvement in the NPV values.

Extrapolating the conditions of simple urban forms to the complex urban morphologies that can be observed in current cities is not commendable. However, aggregated urban forms based on simple urban forms can provide an indication regarding the behavior of complex urban morphologies. An aggregated urban form shows a different behavior than the simple modular forms that were used to build them up. More importantly, no significant difference can be observed among them regarding their energy demand, renewable energy integration and energy system design. This indicates that the influence of the urban form is trivial, whenever we introduce a neighborhood into a highly dense large city, whose complexity is not altered by this introduction. However, the neighborhood configuration should be carefully considered when introducing a stock of buildings at the periphery of the city or in the suburbs, as this new configuration can notably influence the energy performance of the neighborhood. More importantly, this study revealed that the impact of urban forms on the energy system regarding cost is larger than their impact on the annual energy or peak demand. Therefore, it is difficult to deduct the influence of urban forms simply through building and/or urban energy simulations. However, the urban form influences the energy system with regard to cost, system autonomy and renewable energy integration. This makes it essential to optimize the a neighborhood configuration along with the energy system and to combine urban planning and energy system optimization for the future energy transition of cities. However, we need to understand that the uncertainties due to climate conditions, building refurbishment, and cost of energy technologies can notably influence the results quantitatively^11,12,26^.

### Methodology

A computational platform consisting of several computational tools was employed to design urban energy systems for each archetype: an overview of the latter is given in ref. ^11,13^. The platform includes a GIS database (QGIS), comprising the building location, building height, year of construction, etc., a parametric modelling tool (Rhinoceros 5) and an urban simulation model (CitySim). CitySim is well described in literature^11,27,28^ and was validated with on-site monitoring and procedure tests^29,30^. Hourly electricity, heating and cooling demands, as well as solar electricity generation using rooftop BiPV can be computed using this software.

A multi energy hub that caters the heating, cooling and electricity demand of the location is considered in this study. The energy hub is operated in a grid connected mode both selling and purchasing electricity to and from the grid, while accommodating fluctuations in demand and generation. Grid curtailments are introduced when interacting with the grid for purchasing and selling electricity to stabilize the grid. The energy hub consists of renewable energy technologies, a dispatchable energy source and an energy storage facility. Solar photovoltaic power plants and wind turbines are considered as renewable energy technologies, which are non-dispatchable. An Internal Combustion Generator (ICG) is used as the dispatchable energy source, contributing to absorb the fluctuations in demand along with the battery bank. Heating and cooling demands are catered using reversible heat pumps.

The building envelope is fitted with e-coated insulated glazing and presents an averaged glazing ration of 20% without differentiation of facade orientation. The glazing U-value is equal to 1.2 W·m^−2^K^−1^ and the g-value to 0.5. The U-value of the envelope is defined according to the CitySim and Lesosai database^31^. All buildings are equipped with heating and cooling systems with an indoor air temperature set-point of 20 °C for heating and 26 °C for cooling. The buildings are considered to be residential; consequently the occupancy, lighting and appliance profiles are applied according to national and international normative^30,31^. The houly electricity profiles required for lighting and appliances are adapted from^32,33^. The lighting power density corresponds to 5 W·m^−2 ^^33^; the power density of electrical appliances is equal to 2 W·m^−2 ^^33^.

### Case studies

The energy and electrical simulation models were applied for two climatic conditions: the warm climate of Dubai (United Arab Emirates) and the temperate climate of Hemberg (Switzerland). The weather data used for the analyses was created with the software Meteonorm^34,35^ for a Typical Meteorological Year (TMY), based on the average solar irradiance of the period 1991–2010 and the average ambient temperature of the period 2000–2009. The village of Hemberg (47°18′N, 9°10′E, 935 m asl, Cumulative Solar Irradiance: 1,165 kWh·m^−2^, Heating Degree Days: 4,044) presents a Cfb climate (C: warm temperate; f: fully humid; b: warm summer) according to the Köppen-Geiger classification^36^. During wintertime, the lowest temperature recorded during the month of January is equal to −12.1 °C. The summer is quite warm, with a maximal temperature of 29.1 °C during the month of July, and an average ambient temperature of 16.3 °C. The wind speed is constant throughout the year with an average wind speed of 3 m·s^−1^. The precipitations are quite high with a total of 1,018 mm per year. All the monthly data are summarized in the Supplementary Document.

The city of Dubai (25°16′N, 55°20′E, 0 m asl, Cumulative Solar Irradiance: 1,997 kWh·m^−2^, Cooling Degree Days: 6,196) is characterized by a BWh climate (B: arid; W:desert; h:hot) corresponding to a hot desert climate^36^. The maximal ambient temperature reaches 45 °C during the month of July, while the average temperature during summer time is equal to 35 °C and 20 °C during winter time. Precipitations are mostly absent during the year, just a few events being recorded during winter time, with less than 40 mm of annual rain per. The relative humidity is high and the wind speed limited to 3.7 m·s^−1^.

### Energy system design

A multi energy hub that caters the heating, cooling and electricity demand of the location is considered in this study. The energy hub is operated in a grid connected mode both selling and purchasing electricity to and from the grid while accommodating fluctuations in demand and generation. Grid curtailments are introduced when interacting with the grid for purchasing and selling electricity to stabilize the grid. The energy hub consists of renewable energy technologies, a dispatchable energy source and energy storage facility. Solar PV and wind turbines, which are non-dispatchable, are considered as renewable energy technologies. An Internal Combustion Generator (ICG) is used as the dispatchable energy source, contributing to absorb the fluctuations in demand along with the battery bank. Heating and cooling demands are catered using reversible heat pumps.

### Renewable power generation model

Renewable power generation depends on the corresponding energy potential and installed capacity. Both renewable energy potential and energy conversion systems need to be modeled in order to assess their power generation. This is made in two different steps in this study: firstly, the potential for building integrated solar photovoltaic (BiPV) generation is determined using the building simulation model CitySim, where complex geometries of facades and roofs can be considered. Secondly, due to the difficulty to accommodate complex orientations within the energy systems optimization model, solar PV panels installed on flat roofs and ground mounted panels are considered in the energy system optimization.

Hourly global solar irradiation on a horizontal plane is introduced within the energy system model, to compute the solar irradiation on a tilted plane using an isotropic model. A comprehensive description of the model can be found in ref. ^23,37^. Based on the solar irradiation impinging on the solar PV panels, the energy conversion efficiency of the PV modules ${\eta }_{pv}(t$) at time step t is determined using Eq. 1.

1 $${\eta }_{pv}(t)=p\,[q\frac{{G}_{\beta }}{{G}_{\beta ,0}}+{(\frac{{G}_{\beta }}{{G}_{\beta ,0}})}^{m}]\,[1+r\frac{{\theta }_{cell}}{{\theta }_{cell,0}}+s\frac{AM}{A{M}_{0}}+{(\frac{AM}{A{M}_{0}})}^{u}]$$

AM and θ_cell_ are resp. representing the air mass value and the solar cell temperature. The reference values for G_β0_, θ_cell,0_ AM_0_ are G_β0_ = 1000 Wm^−2^, θ_cell,0_ = 25 °C, AM_0_ = 1.5. Similarly the reference values for p, q, r, s, m, u for different solar PV technologies are taken from ref. ^38^.

The power generated by the solar panels P_SPV_(t) can be calculated using Eq. 2, where η_C-SPV_ represents other minor losses of the energy conversion process of the solar panels.

2 $${{\rm{P}}}_{{\rm{SPV}}}({\rm{t}})={{\rm{G}}}_{{\rm{\beta }}}({\rm{t}})\,{{\rm{\eta }}}_{{\rm{pv}}}({\rm{t}})\,{\rm{A}}\,{{\rm{N}}}_{{\rm{SPV}}}{{\rm{\eta }}}_{C-\mathrm{SPV}}$$

Similar to the solar power generation, the wind power generation is computed using hourly wind speed data obtained from meteorological data-bases^37^. Wind speed at the height of 10 m (provided by the meteorological data bases) is converted for hub level considering the atmospheric boundary layer. Similar to solar PV panels, the power generation through a wind turbine is determined using Eq. 3. A cubic spline interpolation is used to represent

the power generation of the wind turbine.

3 $${P}_{w}(t)=\{\begin{array}{l}\begin{array}{ll}{P}_{w}=0, & \,{v}_{hub}(t) < {v}_{ci}\,\\ {P}_{w}={a}_{1}{{v}_{hub}}^{3}+{b}_{1}{{v}_{hub}}^{2}+{c}_{1}{v}_{hub}+{d}_{1}, & \,{v}_{ci} < {v}_{hub}(t) < {v}_{1}\\ {P}_{w}={a}_{2}{{v}_{hub}}^{3}+{b}_{2}{{v}_{hub}}^{2}+{c}_{2}{v}_{hub}+{d}_{2}, & \,{v}_{1} < {v}_{hub}(t) < {v}_{2}\end{array}\\ \,\,\,\,\,\,\ldots \,\ldots \,\ldots \,\ldots \,\ldots \,\ldots \,\ldots \,\ldots \,\ldots \,\ldots \,\ldots \,\ldots \,\ldots \,\ldots \,\ldots \,\ldots \\ \begin{array}{ll}{P}_{w}={a}_{{n}_{s}}{{v}_{hub}}^{3}+{b}_{{n}_{s}}{{v}_{hub}}^{2}+{c}_{{n}_{s}}{v}_{hub}+{d}_{{n}_{s}}, & \,{v}_{{n}_{s}-1} < {v}_{hub}(t) < {v}_{{n}_{s}}\\ {P}_{w}={P}_{r}, & \,{v}_{r} < {v}_{hub}(t) < {v}_{co}\\ {P}_{w}=0, & \,{v}_{co} < {v}_{hub}(t)\end{array}\end{array}\,$$

v_r_, v_ci_, ${v}_{co}\,$and P_r_ are respectively the rated wind speed, the cut-in wind speed, the cut-off wind speed and the rated power of the wind turbine. In addition, a_i_, b_i_, c_i_, and d_i_ are the coefficients of the polynomial function describing the power curve.

Finally, the power generation of the wind turbines is computed using Eq. 4.

4 $${P}_{w}(t)={P}_{w}(t){N}_{w}{\eta }_{w-inv}$$

where N_w_ denotes the number of wind turbines, which is determined by the optimization procedure, and η_w-inv_ denotes the DC/AC inverter efficiency.

### Modeling dispatchable energy source, storage and dispatch strategy

Dispatchable energy sources and energy storage facilities assist the urban energy system to withstand the fluctuations in renewable energy generation and demand. Power generation through dispatchable energy sources, the interactions with grid and storage are determined by the dispatch strategy. A bi-level dispatch strategy is used in this study to assist the energy flow^6^. The primary level of the strategy determines the power generation of the Internal Combustion Generator (ICG). Price of grid electricity, state of charge of the battery bank, renewable energy generation and demand are taken as the inputs. The fuzzy automata theory is used to determine the operating load factor of the ICG. The interactions with the grid and energy storage are assessed in the second stage. The finite automata theory is used to determine the energy flow. A detailed description of the dispatch strategy is given in ref. ^35^. The lifetimes of the ICG and battery bank are determined based on their usage. The life time of the battery bank is determined using the number of charge-discharge cycles and depth of discharge. The lifetime of the ICG is determined using operating hours. Fuel consumption of the ICG is determined based on the operating load factor (LF) of the ICG according to Eq. 5.

5 $$FC=\mathop{\sum }\limits_{t=1}^{8760}({a}_{r,0}+{a}_{r,1}\,LF\,(t)+{a}_{r,2}\,L{F}^{2}(t)+{a}_{r,3}\,L{F}^{3}(t)+\,{a}_{r,4}\,L{F}^{4}(t))$$

where a_r,0_, a_r,1_, a_r,2_, a_r,3_, and a_r,4_ for each ICG are depending from its performance curve.

### Objective functions and optimization constraints

A Pareto optimization was conducted in this study to handle with conflicting multi-objectives by using two objective functions. Optimal solutions results in a Pareto front which presents a set of non-dominant solutions considering the two objective functions. Net-Present Value (NPV) and Grid Integration level (GI) are considered in the objective functions. NPV and GI give a sound overview of the financial aspect and autonomy level of the energy system. Power supply reliability is considered as a constraint to be satisfied while catering the energy demand.

NPV represents the initial capital cost (ICC) for the system components and the recurrent cash flows. ICC includes purchasing and installation cost for system components. Recurrent cash flows includes the system operation and maintenance cost (OM) as well as the grid integration cost: OM can be subdivided into a fixed OM (FOM) and a variable OM (VOM). FOM considers the regular maintenance costs for renewable energy technologies as well as the ICG. Cash flows due to replacement of the battery bank or the ICG are considered within the VOM. Finally, NPV can be formulated according to Eq. 6.

6 $$NPV=ICC\,+\sum _{\forall s\in S}(FO{M}_{s}\,CR{F}_{s})+\mathop{\sum }\limits_{l=1}^{h}\sum _{\forall s\in S}{p}^{l}VO{M}_{s,l}+CRF\,\mathop{\sum }\limits_{t=1}^{t=8760}(PFG(t)\,GCF(t)+PTG(t)\,GCT(t))$$

where ELD(t) represents the energy demand during the steady state operation in time step t. In Eq. 6, CRF, PFG, PTG, GCF, GCF denote capital recovery factor, energy units purchased from grid, energy units sell sold to grid as well as cost of energy in the grid for purchasing and selling respectively.

According to ref. ^6^, the autonomy level of the energy system can be formulated in different ways. This study uses the Grid Integration level (GI) as the indicator for autonomy following ref. ^5^. The GI is defined according to Eq. 7.

7 $$GI=\frac{\mathop{\sum }\limits_{t=1}^{8760}PFG(t)}{\mathop{\sum }\limits_{t=1}^{8760}ELD(t)}$$

In this equation ELD denotes the electricity load demand. The loss of load probability (LOLP) is considered as a constraint in the optimization problem. Loss of power supply LPS (t) for time step t can be computed using Eq. 8 for 8760 time steps (24 × 365). Subsequently, LOLP can be computed according to Eq. 9.

8 $${\rm{LPS}}({\rm{t}})={\rm{ELD}}({\rm{t}})-{{\rm{P}}}_{{\rm{RE}}}({\rm{t}})-{{\rm{P}}}_{{\rm{ngen}}}({\rm{t}})-{{\rm{P}}}_{{\rm{Bat}}\mbox{--}{\rm{Max}}}({\rm{t}})-{{\rm{P}}}_{{\rm{FG}}\mbox{--}{\rm{Max}}}({\rm{t}})$$

where ELD, P_ngen_, P_Bat-Max_ and P_FG-Max_ are the electricity load demand of the application, the ICG nominal power, the maximum power flow from the battery depending on the state of charge, and the maximum power that can be taken from the grid considering grid curtailments. Finally, the loss of load probability (LOLP), calculated using LPS according to Eq. 9, is used as the performance indicator to evaluate the power supply reliability.

9 $$LOLP=\frac{\mathop{\sum }\limits_{t=1}^{8760}LPS(t)}{\mathop{\sum }\limits_{t=1}^{8760}ELD(t)}$$

Different techniques have been used to optimize distributed energy systems. This study uses the method suggested by Perera *et al*.^6,39^. Both energy system design and dispatch strategy are considered in the optimization process. A detailed description of the optimization procedure can be found in ref. ^6^.

## Supplementary information

Supplementary Information
