# Supplementary material for: The influence of urban form on the grid integration of renewable energy technologies and distributed energy systems
Source: Sci Rep. 2019 Nov 28;9:17756. doi: 10.1038/s41598-019-53653-w (PMC6882853; doi:10.1038/s41598-019-53653-w)
Supplement: Supplementary file 1 — Supplementary Information [file 41598_2019_53653_MOESM1_ESM.pdf]

The influence of urban form on the grid integration of renewable energy technologies and distributed energy systems

A.T.D. Perera[1], Silvia Coccolo , Jean-Louis Scartezzini

Solar Energy and Building Physics Laboratory (LESO-PB), Ecole Polytechnique Fédérale de Lausanne (EPFL), CH-1015 Lausanne, Switzerland

SUPPLEMENTARY INFORMATION

Weather conditions in the two cities used for the case study

All the monthly data explaining the weather conditions data are summarized in Figures 1 and 2.

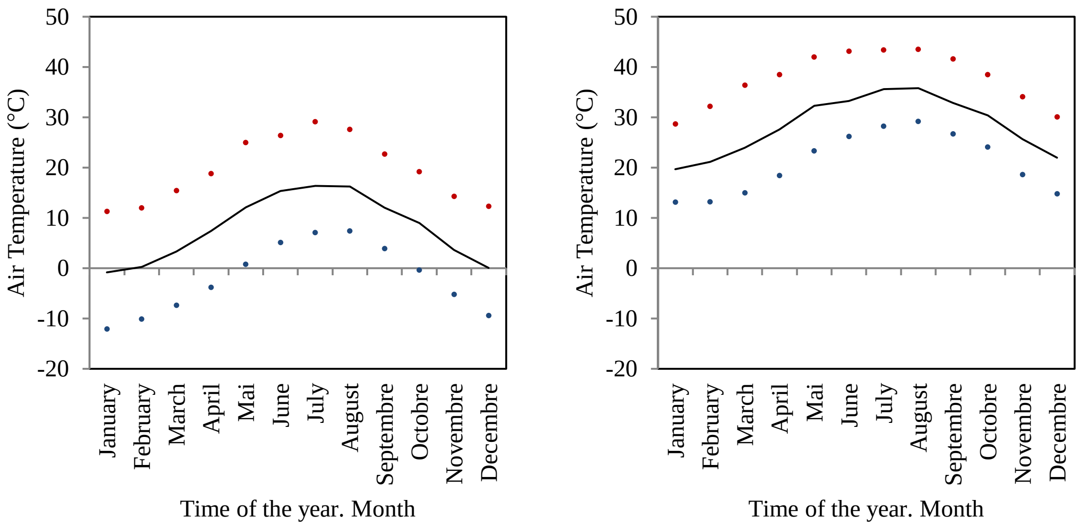

Fig. A1 Monthly averaged air temperature (°C) in the city of Hemberg (left) and Dubai (right).

[1] Corresponding Author
Email:dasun.perera@epfl.ch,
Tel: +41 21 69 35746, Fax: +41 21 693 2722

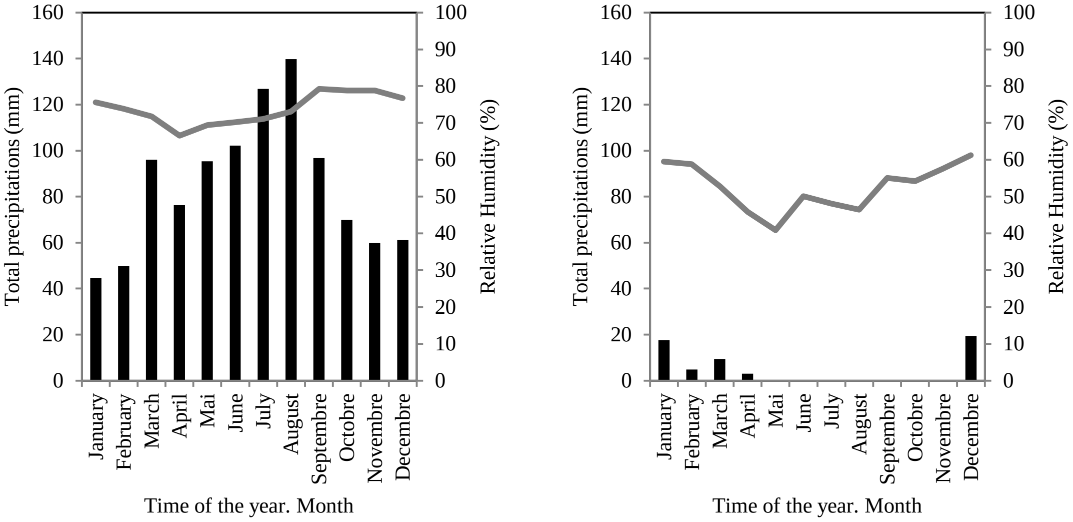

Fig. A2 Monthly averaged relative humidity (%) and total precipitations (mm) in the city of Hemberg (left) and Dubai (right).

Tables A1 and A2 summarize the geometrical properties of each case study.

Characteristics of the archetypes considered in this study

Tables A1- A2, Figure A3 summarise the urban characteristics of each case study.

Table A1 Urban characteristics of each case study, from A to C. Detailed geometrical data.

District	Case Study	Number of Floors	Floor Area (m^2)	Floor Area Ratio (-)	Form Factor (-)	SkyView Factor (building)	SkyView Factor (ground)
A	1	1	900	0.009	2.30	0.60	1.00
	2	2	1,800	0.018	1.55	0.59	0.96
	3	3	2,700	0.027	1.30	0.59	0.93
	4	4	3,600	0.036	1.18	0.59	0.90
	5	5	4,500	0.045	1.10	0.59	0.88
	6	6	5,400	0.054	1.05	0.58	0.87
	7	7	6,300	0.063	1.01	0.58	0.85
	8	8	7,200	0.072	0.99	0.58	0.84
	9	9	8,100	0.081	0.97	0.58	0.83
	10	10	9,000	0.09	0.95	0.57	0.82
B	1	1	3,000	0.03	3.13	0.66	0.96
	2	2	6,000	0.06	1.65	0.65	0.91
	3	3	9,000	0.09	1.25	0.64	0.84
	4	4	12,000	0.12	0.61	0.55	0.66
	5	5	15,000	0.15	0.56	0.55	0.65
	6	6	18,000	0.18	0.53	0.55	0.64
	7	7	21,000	0.21	0.67	0.56	0.67
	8	8	24,000	0.24	0.65	0.55	0.64
	9	9	27,000	0.27	0.63	0.55	0.62
	10	10	30,000	0.3	0.61	0.55	0.60
C	1	1	1,500	0.015	2.29	0.66	0.93
	2	2	3,000	0.03	1.40	0.66	0.86
	3	3	4,500	0.045	1.11	0.66	0.82
	4	4	6,000	0.06	0.74	0.55	0.79
	5	5	7,500	0.075	0.69	0.55	0.77
	6	6	9,000	0.09	0.66	0.54	0.75
	7	7	10,500	0.105	0.73	0.56	0.74
	8	8	12,000	0.12	0.71	0.56	0.72
	9	9	13,500	0.135	0.69	0.56	0.71
	10	10	15,000	0.15	0.67	0.56	0.70

Table A2 Urban characteristics of each case study, from D to F. Detailed geometrical data.

District	Case Study	Number of Floors	Treated Floor Area (m^2)	Floor Area Ratio (-)	Form Factor (-)	SkyView factor (building)	SkyView factor (ground)
D	1	1	4,050	0.0405	2.05	0.71	0.97
	2	2	8,100	0.081	1.23	0.70	0.91
	3	3	12,150	0.1215	1.04	0.69	0.82
	4	4	16,200	0.162	0.88	0.68	0.74
	5	5	20,250	0.2025	0.79	0.67	0.67
	6	6	24,300	0.243	0.72	0.66	0.62
	7	7	28,350	0.2835	0.68	0.65	0.57
	8	8	32,400	0.324	0.59	0.66	0.56
	9	9	36,450	0.3645	0.57	0.65	0.53
	10	10	40,500	0.405	0.55	0.65	0.51
E	1	1	3,000	0.03	1.90	0.68	0.97
	2	2	6,000	0.06	1.15	0.67	0.91
	3	3	9,000	0.09	0.90	0.66	0.86
	4	4	12,000	0.12	0.78	0.65	0.81
	5	5	15,000	0.15	0.70	0.64	0.78
	6	6	18,000	0.18	0.65	0.63	0.75
	7	7	21,000	0.21	0.62	0.61	0.72
	8	8	24,000	0.24	0.59	0.61	0.70
	9	9	27,000	0.27	0.57	0.61	0.69
	10	10	30,000	0.3	0.55	0.61	0.68
F	1	1	5,100	0.051	1.90	0.65	0.96
	2	2	10,200	0.102	1.15	0.64	0.88
	3	3	15,300	0.153	0.90	0.63	0.79
	4	4	20,400	0.204	0.78	0.62	0.71
	5	5	25,500	0.255	0.70	0.61	0.65
	6	6	30,600	0.306	0.65	0.60	0.60
	7	7	35,700	0.357	0.61	0.60	0.56
	8	8	40,800	0.408	0.59	0.59	0.53
	9	9	45,900	0.459	0.57	0.58	0.51
	10	10	51,000	0.51	0.55	0.58	0.49

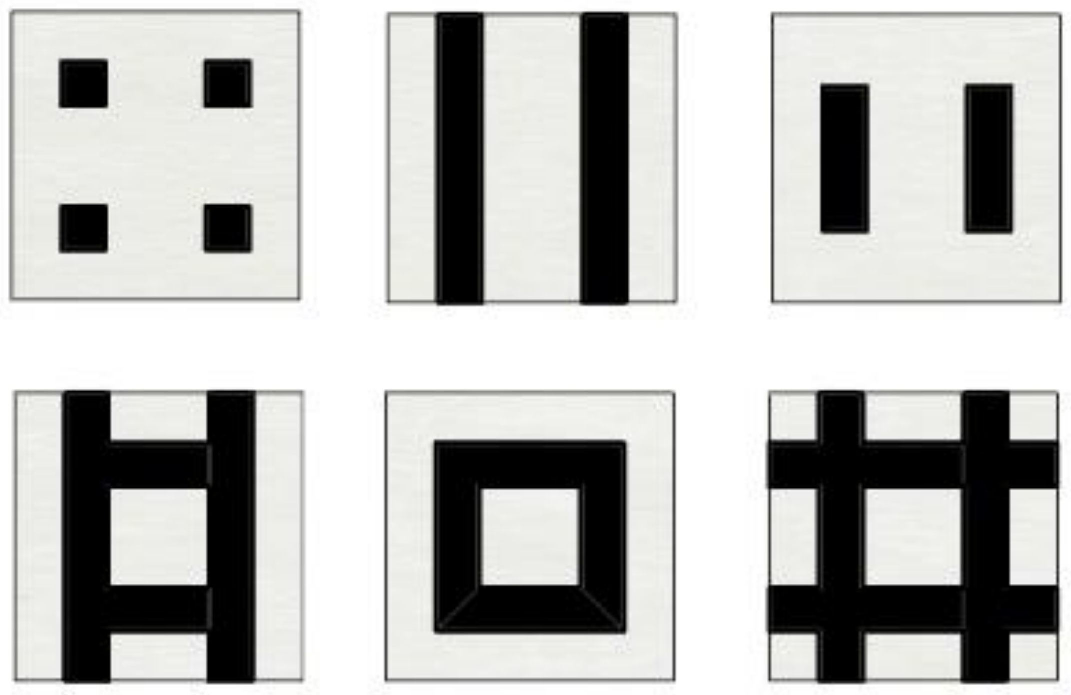

Fig. A3 Six selected archetypes, plan view

The conceptual design of the complex urban forms, starting from the modular archetypes is presented in Figure A4.

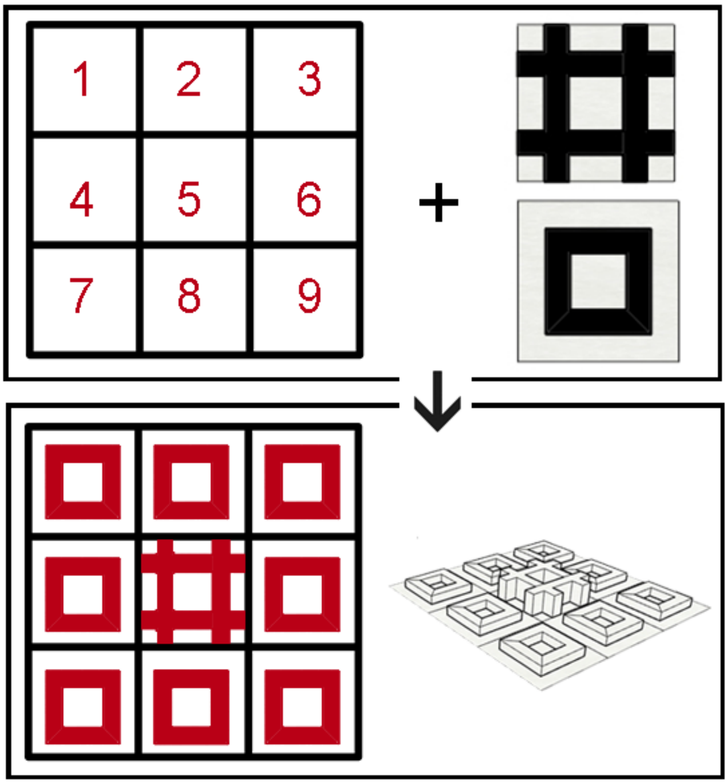

Fig. A4 Conceptual design of the complex urban forms.
